# Comparative Analysis of Industrial Fused Magnesia from Natural and Flotation-Processed Magnesite: Associations Among CaO/SiO_2_ Ratio, Silicate Phase Formation, and Microcracking

**DOI:** 10.3390/ma19030463

**Published:** 2026-01-23

**Authors:** Chunyan Wang, Jian Luan, Zhitao Yang, Qigang Ma, Gang Wang, Ximin Zang

**Affiliations:** 1 School of Materials and Metallurgy, University of Science and Technology Liaoning, Anshan 114051, China; chunyanwang0310@163.com (C.W.); yangzt0527@163.com (Z.Y.); 2 Gansu Runyuan Environmental Resource Technology Co., Ltd., Jiuquan 735000, China; kdcyxe@yeah.net; 3 State Key Laboratory of Advanced Refractories, Luoyang 471039, China; 4 Shenyang University of Technology, Shenyang 121001, China

**Keywords:** magnesite, impurity occurrence, flotation beneficiation, fused magnesia, CaO/SiO_2_ ratio

## Abstract

**Highlights:**

**What are the main findings?**

- Reverse flotation coincided with higher CaO/SiO_2_ ratio (0.68 to 2.85).
- Higher CaO/SiO_2_ coincided with C_2_S dominance; a lower ratio coincided with CMS.
- More C_2_S coincided with more microcracks and slightly lower densification.

**What are the implications of the main findings?**

- Beneficiation may benefit from controlling oxide balance, not only impurities.
- Tailoring CaO/SiO_2_ may help avoid conditions linked to C_2_S and microcracking.
- The findings are derived from industrial comparison; causality requires controlled experiments.

**Abstract:**

In view of the depletion of high-grade magnesite resources in China, this study presents a comparative analysis of two industrial fused magnesia products produced via a flotation–fusion route. A low-grade magnesite (DSQLM-3, MgO 41.48 wt.%) was upgraded by reverse flotation to a concentrate (FDSQLM-3, MgO 47.55 wt.%) with >97% SiO_2_ removal. Two fused magnesia samples (FM-1 from natural high-grade ore DSQLM-1; FFM-3 from concentrate FDSQLM-3) were produced under identical arc-furnace melting (2800 °C, 4 h), followed by natural cooling. Although FFM-3 showed higher MgO (97.61 vs. 97.25 wt.%), its bulk density was comparable to FM-1 (3.45 vs. 3.46 g/cm^3^). XRD/Rietveld refinement and SEM-EDS indicated that CMS dominated the Ca–silicate assemblage in FM-1, whereas β/γ-C_2_S was observed in FFM-3, coinciding with a higher CaO/SiO_2_ (C/S) ratio (2.85 vs. 0.68). Image analysis further showed higher grain boundary microcrack metrics in FFM-3. These observations are consistent with reports in the literature stating that the β → γ transformation of C_2_S during cooling involves ~12% volume expansion that can contribute to cracking; however, cooling history and composition were not independently controlled in this industrial comparison, so the relationships are interpreted as data-supported associations rather than isolated causality. The results suggest that beneficiation strategies may benefit from managing residual oxide balance (especially C/S ratio) in addition to reducing total impurities. Mechanical and thermomechanical properties were not measured and should be evaluated in future work.

## 1. Introduction

As one of the world’s most important mineral resources, magnesite plays an essential role in the production of high-value products, especially fused magnesia, which serves as a critical raw material for advanced refractory linings in metallurgical applications. These include ladles and refining furnaces where exceptional thermal stability is required [1,2,3,4,5,6,7]. However, with the gradual depletion of high-grade magnesite reserves (MgO concentration > 46%), there is a growing need to develop efficient utilization pathways for medium- and low-grade ores (MgO concentration < 43%) to support the sustainable development of the refractory industry [8,9,10].

Magnesite is commonly associated with carbonate minerals, namely dolomite (CaMg(CO_3_)_2_) and calcite (CaCO_3_) [11,12,13], as well as silicate minerals such as quartz, talc, and chlorite. These associated minerals introduce notable levels of impurities, mainly CaO, SiO_2_, Fe_2_O_3_, and Al_2_O_3_. During calcination or fusion, SiO_2_ tends to form low-melting-point silicate phases, and concurrently, CaO can react with SiO_2_ to form β-C_2_S, which is reported to undergo a β → γ transformation upon cooling, involving volumetric expansion that may contribute to cracking/disintegration in Ca-bearing systems [14,15]. Such impurity-induced reactions markedly worsen the high-temperature performance and service life of refractory materials [16,17].

Flotation, as an efficient mineral separation method, has been widely employed in the purification of magnesite ores [18,19,20]. Current research efforts are primarily devoted to reverse flotation for silica removal and direct flotation for the reduction of calcium content, with extensive investigations into the separation mechanisms between magnesite and associated gangue minerals such as dolomite, quartz, and chlorite [21,22,23,24,25]. Previous studies have shown that the mineralogical characteristics of these gangue minerals considerably complicate the beneficiation process [26,27,28,29,30,31]. In particular, the similar surface properties and solubility of magnesite and gangue minerals often result in poor flotation selectivity, ultimately influencing the grade and quality of the concentrate. Although flotation concentrates are increasingly used in the production of fused magnesia, residual gangue components and flotation reagents may negatively impact the final product performance [32]. However, few studies have focused on a systematic comparison of the performance and microstructure between fused magnesia derived from flotation concentrates and natural high-grade ores. Moreover, the mechanisms through which raw material purity and impurity distribution influence periclase (MgO) grain growth, grain boundary characteristics, and secondary-phase precipitation during the fusion process remain inadequately understood.

In this study, representative high-, medium-, and low-grade magnesite ores from the Dashiqiao region (Liaoning, China) were selected to investigate the occurrence and distribution of impurities, and their implications for beneficiation and fused magnesia production. The low-grade ore (DSQLM-3) was upgraded by reverse flotation to obtain a concentrate (FDSQLM-3), and both this concentrate and a natural high-grade ore (DSQLM-1) were used to prepare fused magnesia under identical fusion conditions. Given the industrial origin and processing conditions of the samples, where variables such as composition and cooling history are inherently coupled, this study is positioned as a comparative analysis of two industrial material streams. The aim is to identify significant associations between impurity characteristics (especially the CaO/SiO_2_ (C/S) ratio) and the resulting microstructure, rather than to isolate singular causal mechanisms.

By combining chemical analysis, density measurements, XRD, and SEM–EDS, this work compares how differences in impurity occurrence (intergranular, intragranular, and solid solution impurities) and residual oxide balance (particularly the C/S ratio) are associated with differences in Ca–silicate phase composition (CMS vs. C_2_S) and microcrack characteristics in the two fused magnesia products. This study also examines the impact of these impurities on densification behavior and microstructural integrity. Compared with previous studies that mainly emphasize flotation separation mechanisms or impurity removal efficiency, the present work provides a direct correlation between impurity occurrence states and flotation removability, demonstrating that selective silica removal can significantly increase the C/S ratio and shift the dominant impurity phase from CMS to C_2_S. Moreover, it offers a side-by-side comparison of fused magnesia derived from flotation concentrate and natural high-grade ore under identical processing conditions.

These findings suggest that, in addition to impurity removal, controlling impurity composition, particularly by tailoring the C/S ratio, is crucial for the high-value utilization of low-grade magnesite in refractory applications. However, it should be noted that the cooling history and composition were not independently controlled, and thus, the observed relationships should be interpreted as data-supported associations, not as causal inferences. Future studies should consider verifying these findings through controlled experiments to isolate the effects of specific variables.

## 2. Materials and Methods

### 2.1. Raw Materials

The magnesites used in this work were obtained from representative deposits in the Dashiqiao region, Liaoning Province, China. Based on the MgO concentration in the ores, the samples were categorized into high-, medium-, and low-grade groups and were designated as DSQLM-1, DSQLM-2, and DSQLM-3, respectively. Representative sampling was performed using the “lump sampling method,” with original particle sizes ranging from 30 to 120 mm to ensure sample representativeness. To increase the purity of the DSQLM-3 sample, the ore was crushed and ground to a flotation feed of 100% passing 0.088 mm (−88 μm). The flotation feed was selected by screening through a 200 mesh sieve, and the undersized material was used for flotation. Due to some inherent errors in the screening process, the exact granulometric range may not be very precise. Specifically, the feed mainly consisted of particles passing through a 180 mesh sieve (approximately 88 μm), but due to the lack of particle size analysis data, we cannot provide a more precise granulometric distribution. We have clarified this process in Section 2 and included a description of the screening process to help readers understand the sample preparation method.

Regarding the grinding process, the sample was first crushed using a jaw crusher and then ground using standard ball milling methods, with a grinding time of approximately 30 min and a grinding medium to ensure that the sample achieved the desired particle size range for flotation. The sample was then processed as described above, following the reverse flotation procedure in a closed-circuit flowsheet consisting of single roughing, cleaning, and scavenging stages. Flotation was conducted under weakly alkaline conditions (pH 8.5 ± 0.2) at ambient temperature (≈20–25 °C). The pulp density was maintained at approximately 25–30 wt.% solids (controlled by plant water addition). Sodium silicate was used as a combined depressant/dispersant and added prior to the collector, followed by the use of sodium oleate as the collector. To improve reproducibility, the reagent dosages applied during this campaign are reported as operating windows: sodium silicate ≈ 0.2–0.8 kg·t^−1^ and sodium oleate ≈ 0.2–0.4 kg·t^−1^ (adjusted within these ranges according to routine plant practice and feed variability). After each reagent addition, the pulp was conditioned for ~3 min under mechanical agitation, and the flotation residence time was ~3 min under plant-set aeration/agitation conditions (equipment-fixed parameters). The flotation concentrate was washed with clean water to remove residual reagents prior to fusion. The resulting concentrate (FDSQLM-3) was dried at 110 °C for 24 h before smelting experiments.

### 2.2. Preparation of Fused Magnesia

The raw ore DSQLM-1 and the flotation concentrate FDSQLM-3 were selected to prepare fused magnesia. They were first calcined at 900 °C for 0.5 h in a suspension furnace to obtain light-burned magnesia. The resulting material was subsequently pressed into cylindrical specimens (φ50 × 80 mm). These specimens were then subjected to melting in an industrial arc furnace at 2800 °C for 4 h. After being allowed to cool naturally to room temperature, blocks from the central region of the fused product were selected as representative fused magnesia samples for further analysis, which were free of visible macropores.

### 2.3. Characterization

The elemental compositions of the raw ores, flotation concentrates, and fused magnesia products were determined by X-ray fluorescence spectrometry (XRF, Bruker S8 TIGER, Marlborough, Germany). Phase identification was carried out using X-ray diffraction (XRD, Bruker D8 ADVANCE, Marlborough, Germany) with Cu-Kα radiation. Measurements were performed over a 2θ range from 10° to 90° with a step size of 0.02°. Microstructural features, grain morphology, and localized chemical composition were examined using field emission scanning electron microscopy (FE-SEM, Zeiss SIGMA HD, Oberkochen, Germany), along with an energy dispersive spectroscopy system (EDS, OXFORD INCA 50 mm^2^, OXFORD Instruments, Oxford, UK). The bulk density of the fused magnesia samples was measured in accordance with the Chinese national standard GB/T 2999-2016 [33].

## 3. Results

### 3.1. Physicochemical Properties of Raw Materials

The chemical compositions of the raw ores and the flotation concentrate are summarized in Table 1. All samples are predominantly composed of MgO, with major impurities including SiO_2_, CaO, Al_2_O_3_, and Fe_2_O_3_. DSQLM-1, with an MgO content of 46.92%, qualifies as first-grade magnesite according to industry standards [34]. In contrast, although DSQLM-2 is classified as second-grade based on its MgO concentration, its high SiO_2_ content (3.07%) places it into the category of high-silica magnesite [35,36,37]. DSQLM-3 exhibits an even greater SiO_2_ content of 8.68%, along with elevated concentrations of CaO, Al_2_O_3_, and Fe_2_O_3_, making it unsuitable for direct use in the production of fused magnesia [38,39].

Following the reverse flotation treatment, the MgO content in the upgraded DSQLM-3 concentrate increased from 41.48% to 47.55%, while the SiO_2_ content was significantly reduced from 8.68% to 0.21%, achieving a removal efficiency of 97.6%. Concurrently, the contents of CaO, Al_2_O_3_, and Fe_2_O_3_ impurities decreased from 1.18%, 0.76%, and 0.88% to 0.70%, 0.14%, and 0.29%, respectively. These results demonstrate that silica and alumina impurities can be effectively removed through flotation, whereas calcium and iron bearing components exhibit greater resistance to separation, likely owing to their distinct occurrence states and surface properties.

**Figure 1:**
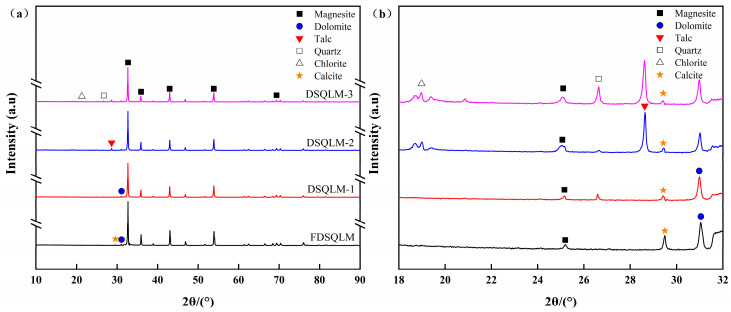
XRD patterns of the raw magnesite ores and identification of major impurity minerals: (**a**) full pattern; (**b**) partial enlargement of 18–32° (black—FDSQLM, red—DSQLM-1, blue—DSQLM-2, and purple—DSQLM-3).

The XRD patterns of the raw ores are presented in Figure 1. The dominant phase detected in all samples was identified as magnesite (MgCO_3_), evidenced by a strong and sharp diffraction peak at 2θ ≈ 32.6°, indicating high crystallinity. DSQLM-1 primarily exhibited peaks corresponding to magnesite, along with a minor dolomite peak at 2θ ≈ 30.9°, consistent with its high chemical purity. In contrast, DSQLM-2 showed a characteristic peak of quartz at 2θ ≈ 26.6°. Additionally, DSQLM-3 displayed distinct diffraction peaks corresponding to talc (2θ ≈ 28.6°) and chlorite (2θ ≈ 12.5°), confirming the presence of complex silicate gangue minerals.

**Figure 2:**
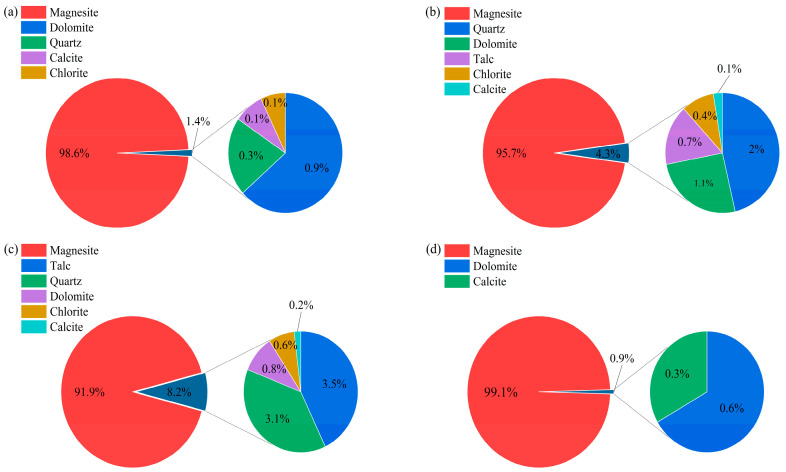
Mineral composition of different magnesites: (**a**) DSQLM-1, (**b**) DSQLM-2, (**c**) DSQLM-3, and (**d**) FDSQLM-3.

The XRD pattern of the flotation concentrate FDSQLM-3 exhibits only trace peaks corresponding to dolomite and calcite, while the diffraction signals associated with quartz, talc, and chlorite are no longer detectable, demonstrating effective removal of silicate-based impurities. Quantitative phase analysis (Figure 2) further confirms that the magnesite content reaches 99.1% in FDSQLM-3, with the concentration of silicate phases reduced to below the detection limit.

Qualitative and semi-quantitative phase analyses were conducted using HighScore software (version 3.0e) to reveal the relative content of each mineral in different ores, as summarized in Figure 2. DSQLM-1 was found to contain magnesite at a level of 98.58%, with total impurities less than 2%. With decreasing ore grade, the contents of quartz, talc, and chlorite increased successively in DSQLM-2 and DSQLM-3. Following the flotation process, the magnesite content in FDSQLM-3 increased to 99.1%—with impurity minerals, particularly silicate phases such as quartz, talc, and chlorite, being effectively eliminated to levels below the detection limit.

### 3.2. Effect of Impurity Occurrence

To elucidate the influence of impurity occurrence on flotation performance, representative magnesite samples (DSQLM-1 to DSQLM-3) were analyzed using FE-SEM coupled with EDS point analysis across 22 regions of interest (Table 2). The EDS point analyses in Table 2 are intended for phase/occurrence identification rather than statistical quantification; points were selected from representative BSE micrographs based on BSE contrast, morphology, and location (grains, fractures, and boundaries), and acquired under consistent instrument conditions. The results reveal significant variations in mineral assemblage, microstructural complexity, and impurity distribution, which collectively govern the efficiency of physical beneficiation processes.

**Figure 3:**
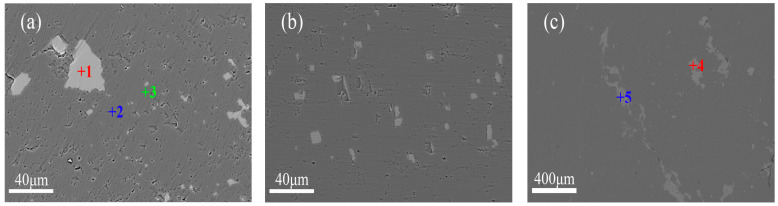
Backscattered electron images of DSQLM-1: (**a**) granular and fine-grained dolomite; (**b**) intragranular dolomite; (**c**) quartz and chlorite at grain boundaries.

As shown in Figure 3, DSQLM-1 exhibits high purity, with magnesite (MgCO_3_) as the dominant mineral phase, typically appearing as granular or irregular crystals with well-developed rhombohedral cleavage. Calcium-bearing impurities are predominantly present in the form of dolomite (CaMg(CO_3_)_2_, either located between magnesite grains with fine sizes (20–40 μm) (Figure 3a) or dissolved within the magnesite matrix as solid solutions (Figure 3b). Owing to the similarities in physical and chemical properties, as well as morphological occurrence between dolomite and magnesite, sufficient liberation through conventional crushing and flotation remains challenging, which is a persistent issue in magnesite beneficiation. Although flotation efficiency has been significantly improved in recent years through the development of novel collectors and combined reagents [40,41,42], finely disseminated inclusions and solid solution calcium-bearing impurities cannot be removed by mechanical processing alone [43,44,45,46,47]. Iron impurities occur mainly in the solid solution form within the magnesite crystal lattice (Point 3), resulting from isomorphous substitution, a form that is not amenable to removal by physical beneficiation methods.

**Figure 4:**
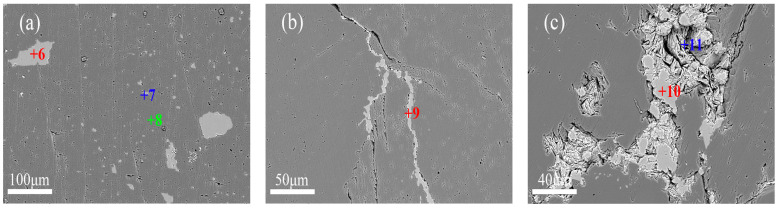
Backscattered electron images of DSQLM-2: (**a**) dolomite; (**b**) banded quartz; (**c**) quartz and chlorite in intergranular pores.

In contrast, DSQLM-2 demonstrates a notably higher gangue content, consistent with the findings from XRD and XRF analyses, as displayed in Figure 4. While the distribution of dolomite remains similar to that observed in DSQLM-1 (Figure 4a), a significant portion of the quartz phase (SiO_2_) occurs as banded morphology infilling intergranular spaces within the magnesite matrix, with individual bands exceeding 100 μm in length (Figure 4b). Gangue minerals in this type can be effectively removed through physical separation methods such as flotation, provided that sufficient liberation is achieved during crushing and grinding [48,49]. Additionally, certain fractions of quartz and chlorite ([(Mg, Fe^2+^, Al)_3_(Si, Al)_4_O_10_(OH)_2_]) occur as granular composites in the cracks and grain boundaries (Figure 4c). Chlorite, a layered silicate mineral, typically forms flaky or aggregated microstructures. Due to its soft and brittle nature, chlorite is prone to generating ultrafine slimes during grinding. These slimes may adhere to/coating magnesite surfaces (slime coating) and/or be mechanically entrained into the froth, thereby reducing flotation selectivity and deteriorating reverse flotation performance (e.g., lower MgO grade and/or higher gangue content in the concentrate, as well as potentially reduced recovery) [50].

**Figure 5:**
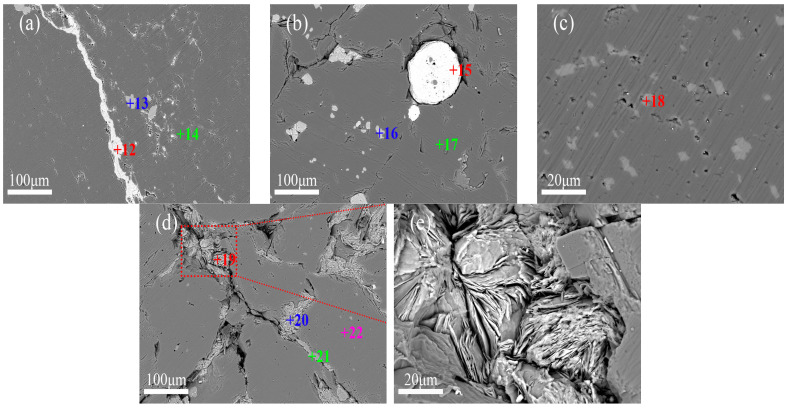
Backscattered electron images of DSQLM-3: (**a**) banded calcite; (**b**) hematite; (**c**) talc, chlorite, and quartz in fractures; (**d**) radiating talc and chlorite; (**e**) fine-grained dolomite.

DSQLM-3, the lowest-grade magnesite ore in this study, exhibits the highest gangue content and the most complex mineralogical assemblage among the three samples. The gangue assemblage includes dolomite, calcite (CaCO_3_), quartz (SiO_2_), chlorite ([(Mg, Fe^2+^, Al)_3_(Si, Al)_4_O_10_(OH)_2_]), talc (Mg_3_Si_4_O_10_(OH)_2_), and minor hematite and fluorapatite (Ca_5_(PO_4_)_3_F). Calcite occurs in banded structures (Figure 5a), while minor hematite forms near spherical particles ranging from 30 to 150 μm, and it can be removed by magnetic separation [51]. Fluorapatite, rarely reported in such ores, occurs as fine-grained (5–40 μm) discrete minerals; its removal is expected to require fine grinding due to its particle size characteristics. Both talc and chlorite, which are layered silicate minerals, form flaky aggregates in the microstructure (Figure 5d). These two minerals can be distinguished by EDS elemental analysis (e.g., Point 19 for talc and Point 20 for chlorite). Notably, talc is naturally hydrophobic and tends to report spontaneously to the flotation concentrate. Similar to chlorite, it is highly susceptible to slime formation during grinding, which adversely affects flotation selectivity and efficiency [50].

**Figure 6:**
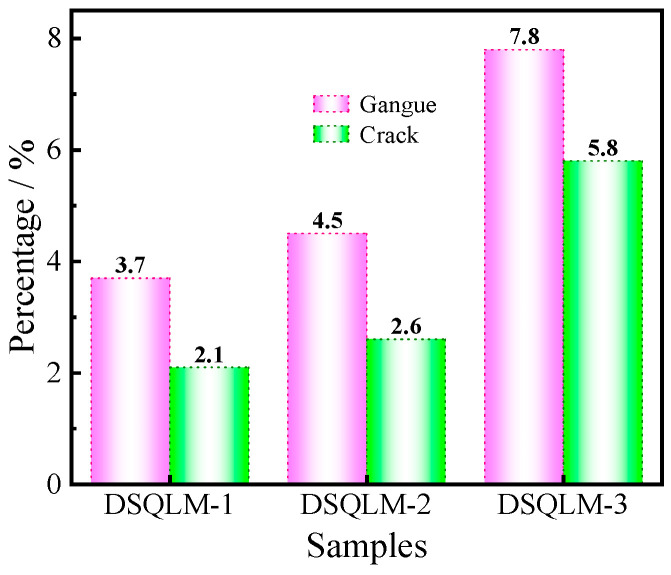
Proportions of gangue and crack in the different magnesite samples.

Quantitative image analysis of backscattered electron (BSE) micrographs (Figure 3c, Figure 4b and Figure 5d) using ImageJ software (version 1.8.0) reveals that gangue minerals and microcracks occupy area fractions of 7.8% and 5.8%, respectively (Figure 6). The elevated porosity arises primarily from the increased interfacial boundaries between gangue minerals and magnesite, as well as among different gangue species, manifesting macroscopically as increased overall ore porosity. Such a microstructure may hinder selective bubble–particle attachment and enhance mechanical entrainment (non-selective carryover of fine particles) in reverse flotation, potentially causing fine magnesite losses to the froth and reducing separation selectivity [52,53,54,55].

### 3.3. Microstructure and Densification Behavior of Fused Magnesia

Fused magnesia samples designated as FM-1 and FFM-3 were produced via a calcination pelletization fusion process using natural high-grade magnesite (DSQLM-1) and flotation-derived concentrate (FDSQLM-3), respectively. Chemical analysis (Table 3) indicates that FFM-3 exhibits a slightly higher MgO content of 97.61 wt.% compared to 97.25 wt.% for FM-1. The total impurity content (ΣCaO + Al_2_O_3_ + SiO_2_ + Fe_2_O_3_) is 2.61 wt.% for FM-1 and 2.24 wt.% for FFM-3 after flotation, confirming the effectiveness of flotation in removing silicate and aluminate gangue minerals. Notably, a significant difference is observed in the CaO/SiO_2_ (C/S) ratio: 0.68 for FM-1 versus 2.85 for FFM-3. This is consistent with selective silica removal being accompanied by a higher C/S ratio (i.e., relative enrichment of CaO with respect to SiO_2_) in the final product.

Additionally, to ensure the accuracy of bulk density measurements, we measured five specimens of FM-1 and FFM-3 fused magnesia samples. The measured mean values and standard deviations of the bulk density are as follows:

FM-1: Bulk density = 3.46 g/cm^3^; standard deviation = 0.01 g/cm^3^;

FFM-3: Bulk density = 3.45 g/cm^3^; standard deviation = 0.01 g/cm^3^.

This minor difference in bulk density (0.01 g/cm^3^) was found to be statistically insignificant based on a *t*-test analysis, confirming that the bulk densities of FM-1 and FFM-3 are effectively comparable.

The CaO/SiO_2_ (C/S) ratio is a critical parameter that determines the equilibrium phase composition in the MgO–CaO–SiO_2_ ternary system [56,57]. It should be noted that the phase diagram interpretation reflects an equilibrium tendency, whereas industrial arc melting and natural cooling are inherently non-equilibrium processes. Therefore, metastable phase formation/retention and cooling-kinetics-controlled transformations may lead to deviations from the equilibrium assemblage. Table 4 summarizes the equilibrium tendency of primary silicate phases in the MgO–CaO–SiO_2_ system; Al_2_O_3_ and Fe_2_O_3_ are treated as dilute here because their contents are low and comparable in FM-1 and FFM-3 (Table 3). As indicated by the phase diagram analysis, a C/S ratio below 1 favors the formation of low-melting-point phases, such as monticellite (CaO·MgO·SiO_2_, CMS). In contrast, a C/S ratio exceeding 2 promotes the precipitation of high-melting-point dicalcium silicate (2CaO·SiO_2_, C_2_S). In this study, FM-1, with a C/S value of 0.68, predominantly contains CMS as the main silicate phase. FFM-3, with a C/S ratio of 2.85, lies within the stability region of C_2_S and tricalcium silicate (C_3_S). It is noteworthy that C_3_S is metastable below approximately 1250 °C during cooling and that it tends to decompose into C_2_S and free CaO (f-CaO), which may induce additional structural defects and degrade performance.

**Figure 7:**
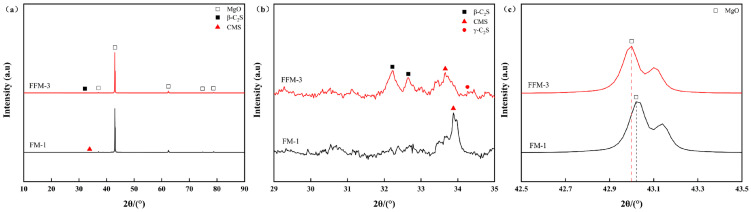
XRD patterns of the two magnesia products: (**a**) full spectrum; (**b**) 2θ range from 29° to 35°; (**c**) 2θ range from 42.5° to 43.5°.

The X-ray diffraction (XRD) patterns presented in Figure 7 indicate that periclase (MgO) constitutes the primary crystalline phase in both fused magnesia samples. A minor phase of calcium magnesium silicate (CMS) is identified in FM-1, whereas distinct diffraction peaks corresponding to dicalcium silicate (C_2_S) are observed in FFM-3. The X-ray diffraction (XRD) patterns presented in Figure 7 indicate that periclase (MgO) constitutes the primary crystalline phase in both fused magnesia samples. A minor phase of calcium magnesium silicate (CMS) is identified in FM-1, whereas distinct diffraction peaks corresponding to dicalcium silicate (C_2_S) are observed in FFM-3. To provide quantitative support, Rietveld refinement and quantitative phase analysis were performed for each sample using HighScore, with MgO (periclase) explicitly included in the refinement. For FM-1, the refinement quality was satisfactory (wRp ≈ 5.75), and the phase fractions were determined to be MgO 95.83 wt.%, CMS 3.12 wt.%, and Ca_3_(PO_4_)_2_ 1.05 wt.%. For FFM-3, the refinement yielded wRp ≈ 6.25, with phase fractions of MgO 98.24 wt.%, β-C_2_S 1.51 wt.%, and γ-C_2_S 0.25 wt.% (Table 5). It should be noted that these values represent the crystalline phases included in the refinement; no internal standard was employed in the present work, and therefore, any amorphous contribution was not quantified. Furthermore, the diffraction peak of the periclase (200 plane) in FFM-3 exhibits a noticeable shift toward lower angles (from 42.952° to 42.931°). This change means an increase in the interplanar spacing (d-value) (from 0.2092 nm to 0.2094 nm), indicating lattice expansion. This phenomenon can be attributed to the partial substitution of Mg^2+^ ions (ionic radius r = 0.072 nm) by larger Ca^2+^ ions (r = 0.100 nm) within the MgO crystal structure, leading to the formation of a (Ca, Mg)O solid solution. This observation aligns with the limited solid solubility of CaO in MgO, as reported by Lampropoulou [57], a process typically facilitated by slow cooling from the high-temperature molten state.

**Figure 8:**
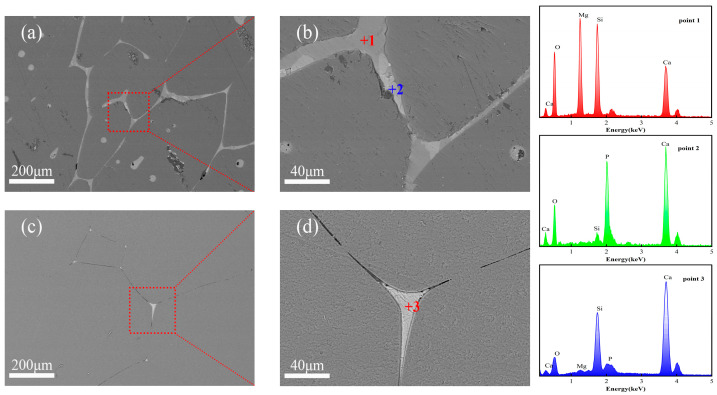
Microstructures of the fused magnesia: (**a**,**b**) FM-1; (**c**,**d**) FFM-3.

Although FFM-3 exhibits higher chemical purity and larger periclase grain sizes (400–600 μm compared to 300–400 μm in FM-1), its bulk density (3.45 g/cm^3^) is slightly lower than that of FM-1 (3.46 g/cm^3^), as shown in Figure 8 and Table 3, suggesting that microstructural integrity rather than chemical or grain growth factors plays a dominant role in the densification of fused magnesia. SEM analysis (Figure 8) shows that FM-1 possesses a uniform and dense microstructure, with grain boundaries and triple points filled by continuously distributed silicate phases. EDS analysis (Table 6) confirms that point 1 corresponds to the CMS phase, and point 2 corresponds to the Ca_3_(PO_4_)_2_ phase. The excellent liquid phase wetting behavior effectively facilitates densification and pore elimination during the final stage of sintering.

In contrast, silicate phases in FFM-3 are significantly reduced and predominantly occur as isolated particles confined to small triangular pores at triple junctions, failing to establish a continuous liquid phase network. More critically, numerous discontinuous and elongated microcracks are observed along the grain boundaries. EDS analysis (point 3) confirms the enrichment of Ca and Si elements in the intergranular silicate phases, corresponding to the presence of β-C_2_S phase observed in the XRD. The β → γ transformation of C_2_S is reported to occur near ~725 °C and involves ~12% volume expansion [58], which could plausibly generate stresses during cooling. In this industrial comparison, the co-occurrence of Ca–Si-enriched regions (consistent with β/γ-C_2_S) and grain boundary microcracking is therefore interpreted as an association consistent with transformation-related cracking scenarios, rather than definitive causal proof.

This observation aligns with the slightly lower bulk density of FFM-3 and suggests potential impacts on microstructural integrity. The implications for mechanical strength and thermal shock resistance are therefore plausible but require direct mechanical/thermomechanical testing for verification.

**Figure 9:**
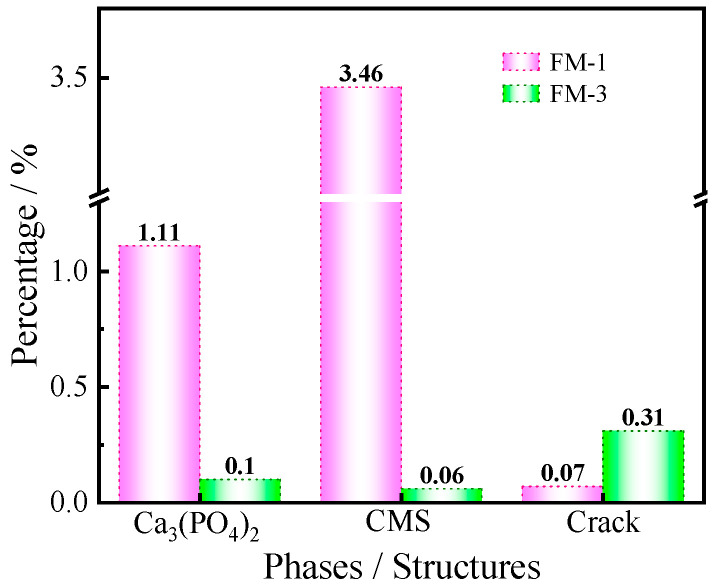
Silicate content and crack area fraction in magnesia samples.

In addition to the qualitative observations, we conducted a quantitative analysis using ImageJ software (Figure 8). The results revealed that in the FM-1 sample, the CMS (calcium magnesium silicate) content was 3.46%, and the Ca_3_(PO_4_)_2_ (tricalcium phosphate) content was 1.11%. For the FFM-3 sample, while the silicate phase content was trace, the crack area fraction was found to be 0.31, significantly higher than the 0.07 observed for FM-1. This higher crack area fraction in FFM-3 suggests increased microstructural damage in the C_2_S-containing sample and may be related to transformation-associated stresses reported for β → γ C_2_S during cooling. Figure 9 illustrates the silicate content and crack area fraction in the magnesia samples, further highlighting the observed differences between FM-1 and FFM-3. The bars in the figure compare the two samples in terms of their respective silicate content and crack area fraction. It is clear from the figure that FM-1 has a much lower crack area fraction compared to FFM-3, with values of 0.07 and 0.31, respectively. This higher crack area fraction in FFM-3 corresponds to increased microstructural damage, which is likely due to the β → γ phase transformation of C_2_S. 

These observations highlight a potential trade-off in the flotation–fusion route—selective silica removal may reshape the residual oxide balance (including C/S ratio), coinciding with the occurrence of Ca-rich silicates such as C_2_S. In the present industrial comparison, C_2_S occurrence coincided with higher microcrack metrics and slightly lower densification, suggesting that concentrate design may benefit from considering oxide balance in addition to total impurity removal. Although flotation effectively reduces the overall impurity content, its selective removal of silica leads to a sharp increase in the CaO/SiO_2_ (C/S) ratio, thereby facilitating the precipitation of the dicalcium silicate (C_2_S) phase. The instability of this phase within the typical service temperature range, particularly under thermal cycling conditions, is due to its polymorphic transformation, accompanied by volumetric change, significantly compromising the structural stability and long-term service performance of refractory products prepared from fused magnesia.

Thus, efficient utilization of low-grade magnesite resources might benefit from a more balanced approach rather than solely focusing on reducing total impurity content. Instead, greater emphasis must be placed on regulating the stoichiometric relationships among residual oxides, particularly through deliberate optimization of the CaO/SiO_2_ (C/S) ratio. By guiding the formation of high-temperature stable, non-transformable intergranular phases such as calcium magnesium silicate (CMS), it becomes possible to achieve both high purity and high performance in fused magnesia products.

Limitations and scope: The comparison of fused magnesia in this study is based on one natural high-grade ore (DSQLM-1 → FM-1) and one flotation-derived concentrate from a low-grade ore (FDSQLM-3 → FFM-3); broader generality should be validated using additional ore sources and multiple concentrates in future work.

## 4. Discussion

The results indicate that the flotation–fusion route is influenced not only by total impurity removal but also by how flotation reshapes the residual oxide balance. Since intergranular silicate gangue is more readily liberated and removed than Ca-bearing impurities occurring as fine intergrown/intragranular dolomite (and Fe partly in solid solution), reverse flotation preferentially depletes SiO_2_ and can be associated with an increase in the CaO/SiO_2_ (C/S) ratio, even when overall purity improves. This shift in the C/S ratio is associated with changes in impurity phase selection: low C/S favors Ca–Mg silicates (CMS), while high C/S is associated with Ca-rich silicates (C_2_S/C_3_S) in the MgO–CaO–SiO_2_ system. While phase diagrams reflect equilibrium tendencies, industrial melting and natural cooling are non-equilibrium processes; thus, metastable retention and cooling-kinetics-controlled transformations may be involved. In this case, the presence of C_2_S in the high-C/S fused magnesia is associated with the β→γ polymorphic transformation near ~725 °C, which involves ~12% volume expansion and may potentially generate internal stresses and grain boundary cracking. This chemistry–phase–damage association is supported by quantitative evidence—HighScore Rietveld-QPA (wRp ≈ 5.75 for FM-1; ≈6.25 for FFM-3) shows CMS (FM-1) versus β/γ-C_2_S (FFM-3) as the dominant Ca–silicate phases, and ImageJ analysis indicates a significantly higher crack area fraction in FFM-3 than in FM-1. SEM–EDS was used here primarily for phase/occurrence identification and localized Ca–Si association with crack/grain boundary regions, rather than for statistical quantification. Overall, the results highlight C/S as a “hidden” control variable in flotation-derived concentrates which may outweigh purity gains by promoting transformation-prone silicates and microcrack formation.

## 5. Conclusions

In this study, the occurrence state and distribution behavior of impurities in different grades of magnesite ores were investigated, and their influence on the flotation efficiency and performance of fused magnesia was also assessed. Based on the results, the following conclusions can be drawn.

(1) The impurities in Dashiqiao magnesite exhibit diverse modes of occurrence. Calcium is predominantly found as dolomite, occurring either as intergranular grains or within crystal grains. Silicon and aluminum are present in silicate minerals, including quartz, talc, and chlorite. Iron substitutes isomorphically within the magnesite lattice in the form of a solid solution, while phosphorus and fluorine are primarily hosted within fluorapatite.
(2) Reverse flotation proves highly effective in removing intergranular silicate impurities, achieving a silica (SiO_2_) removal efficiency exceeding 97%. However, it shows limited efficacy in eliminating intragranular dolomite and iron incorporated in solid solution within the magnesite lattice. This selective removal is associated with the change in the CaO/SiO_2_ (C/S) ratio, which increases from 0.68 to 2.85 after flotation.
(3) In the fused magnesia derived from the flotation concentrate, the elevated C/S ratio is associated with the precipitation of β-C_2_S. Microcrack formation was observed, consistent with the volumetric expansion associated with the β → γ C_2_S transformation during cooling.
(4) The results suggest that for the flotation–fusion route, managing the CaO/SiO_2_ (C/S) ratio in addition to total impurities may help avoid conditions associated with transformation-prone Ca–silicates and potentially improve microstructural stability.

Limitations and scope: The fused-magnesia comparison in this study was based on one high-grade natural ore (DSQLM-1 → FM-1) and one flotation-derived concentrate from a low-grade ore (FDSQLM-3 → FFM-3). While this pairing provides valuable insights into the influence of impurity occurrence and the C/S ratio on phase evolution and microstructure, it introduces a fundamental limitation inherent to industrial comparisons: the inability to independently control key parameters such as the CaO/SiO_2_ ratio and the cooling regime. Consequently, although a strong correlation between a high C/S ratio, C_2_S formation, and microcracking is observed, this study cannot definitively isolate the causative role of each parameter within the proposed mechanism. The findings should therefore be interpreted as identifying critical interlinked factors in an industrial process; the precise mechanistic pathway requires validation through controlled experiments. Furthermore, while we have quantified impurity phase evolution (Rietveld-QPA) and microstructural degradation (ImageJ crack area fraction), more detailed stereological analyses—including crack density/length and full grain-size distributions—were not performed. These metrics, along with direct mechanical/thermomechanical testing, should be explored in future work to better understand the structure–property relationships and to assess the kinetic effects of cooling on the C_2_S polymorphic transformation and microcracking.

## Figures and Tables

**Table 1:** Chemical composition of raw ores and flotation concentrate (wt.%).

Sample NO.	MgO	CaO	Al_2_O_3_	SiO_2_	Fe_2_O_3_	LOI	C/S (n)
DSQLM-1	46.92	0.78	0.48	1.06	0.57	50.19	0.79
DSQLM-2	45.90	0.80	0.06	3.07	0.65	49.52	0.24
DSQLM-3	41.48	1.18	0.76	8.68	0.88	47.02	0.15
F-DSQLM-3	47.55	0.70	0.14	0.21	0.29	51.11	3.57

LOI: loss on ignition.

**Table 2:** EDS composition results of the corresponding phases in Figure 3, Figure 4 and Figure 5.

	Weight Percentage of Elements, wt.%									Phase Composition	Distribution Characteristics	Flotation Difficulty
	Mg	Al	Ca	Si	Fe	C	O	P	F			
1	13.99	—	22.71	—	—	12.24	51.07	—	—	Dolomite	Intergranular granular	Difficult
2	13.52	—	21.28	—	—	13.00	52.20	—	—	Dolomite	Fine-grained intergranular	Very Difficult
3	28.27	—	—	—	0.20	14.35	57.18	—	—	Iron-rich magnesite	Intragranular	Very Difficult
4	—	—	—	45.75	—	—	54.25	—	—	Quartz	Fracture filling	Simple
5	25.87	7.89	—	16.96	—	—	48.39	—	0.89	Fluorine-containing chlorite	Fracture filling	Difficult
6	14.00	—	22.71	—	—	12.24	51.05	—	—	Dolomite	Intergranular granular	Difficult
7	14.19	—	20.35	—	—	13.05	52.41	—	—	Dolomite	Intragranular	Very Difficult
8	28.27	—	—	—	0.19	14.35	57.19	—	—	Iron-rich magnesite	Intragranular	Very Difficult
9	—	—	—	46.70	—	—	53.30	—	—	Quartz	Fracture filling	Simple
10	—	—	—	47.02	—	—	52.98	—	—	Quartz	Fracture filling	Simple
11	18.16	6.71	0.19	16.16	—	5.57	53.21	—	—	chlorite	Fracture filling	Difficult
12	1.69	—	36.06	—	—	12.73	49.52	—	—	Calcite	Fracture filling	Simple
13	—	—	—	46.82	—	—	53.18	—	—	Quartz	Fracture filling	Simple
14	27.48	—	0.53	—	—	14.60	57.39	—	—	Calcium-rich magnesite	—	—
15	—	—	—	—	70.71	—	29.29	—	—	Limonite	Fracture filling	Difficult
16	—	—	28.22	—	—	5.12	45.18	15.70	5.78	Fluorapatite	Fine-grained intergranular	Difficult
17	27.66	—	—	—	—	14.77	57.56	—	—	magnesite	—	—
18	13.81	—	20.51	—	—	13.21	52.47	—	—	Dolomite	Intragranular	Very Difficult
19	15.22	—	—	27.73	—	4.22	52.84	—	—	Talc	Fracture filling	Difficult
20	22.75	8.88	0.27	20.70	—	—	45.56	—	0.84	Fluor–chlorite	Fracture filling	Difficult
21	—	—	—	43.33	—	—	56.67	—	—	Quartz	Fracture filling	Simple
22	27.66	—	—	—	0.19	14.77	57.37	—	—	Iron-rich magnesite	Intragranular	Difficult

**Table 3:** Chemical composition of the fused magnesia (wt.%).

Sample	MgO	CaO	Al_2_O_3_	SiO_2_	Fe_2_O_3_	LOI	B.D. (g/cm^3^)	C/S (n)	Number of Specimens	Statistical Significance
FM-1	97.25	0.73	0.20	1.15	0.53	0.14	3.46	0.68	5	No
FFM-3	97.61	1.09	0.18	0.41	0.56	0.15	3.45	2.85	5	No

B.D.: Bulk density.

**Table 4:** Effect of C/S ratio on the phase composition of magnesia and the melting points of the impurity phases [56].

C/S (n)	Phase Composition and Initial Melting Temperatures of Impurity Phases
<1	MgO, M_2_S (1890), CMS (1495)
1–1.5	MgO, CMS (1495), C_3_MS_2_ (1550)
1.5–2	MgO, C_3_MS_2_ (1550), C_2_S (2130)
>2	MgO, C_2_S (2130)
2–3	MgO, C_2_S (2130), C_3_S (1900)

**Table 5:** Quantitative phase analysis (QPA) results of FM-1 and FFM-3 obtained by Rietveld refinement of XRD data using HighScore.

	MgO	CMS	Ca_3_(PO_4_)_2_	β-C_2_S	γ-C_2_S	wRp
FM-1	95.83	3.12	1.05	—	—	5.75
FFM-3	98.24	—	—	1.51	0.25	6.25

**Table 6:** EDS analysis of points in Figure 8 (wt.%).

	Mg	Si	Ca	P	O	Possible Compositions
1	17.42	18.56	22.46	—	41.57	CMS
2	—	1.35	39.02	18.56	41.07	Ca_3_(PO_4_)_2_
3	1.02	15.01	46.03	0.78	37.16	C_2_S

## Data Availability

The original contributions presented in this study are included in the article. Further inquiries can be directed to the corresponding authors.
